# Genome-Wide Association Study for Multiple Biotic Stress Resistance in Synthetic Hexaploid Wheat

**DOI:** 10.3390/ijms20153667

**Published:** 2019-07-26

**Authors:** Madhav Bhatta, Alexey Morgounov, Vikas Belamkar, Stephen N. Wegulo, Abdelfattah A. Dababat, Gül Erginbas-Orakci, Mustapha El Bouhssini, Pravin Gautam, Jesse Poland, Nilüfer Akci, Lütfü Demir, Ruth Wanyera, P. Stephen Baenziger

**Affiliations:** 1 Department of Agronomy and Horticulture, University of Nebraska, Lincoln, NE 68583, USA; 2 Department of Agronomy, University of Wisconsin-Madison, Madison, WI 53706, USA; 3 International Maize and Wheat Improvement Center (CIMMYT), P.K. 39 Emek, 06511 Ankara, Turkey; 4 Department of Plant Pathology, University of Nebraska-Lincoln, Lincoln, NE 68583, USA; 5 International Center for Agricultural Research in the Dry Areas (ICARDA), Rabat, P.O. Box 6299 Rabat-Institutes, Morocco; 6 BASF Agricultural Solutions Nebraska Research Station, Beaver Crossing, NE 68313, USA; 7 Wheat Genetics Resource Center, Department of Plant Pathology, Kansas State University, Manhattan, KS 66506, USA; 8 Central Field Crop Research Institute, Yenimahalle, 06170 Ankara, Turkey; 9 Maize Research Station, 54060 Sakarya, Turkey; 10 Kenya Agricultural and Livestock Research Organization (KALRO), 90100 Njoro, Kenya

**Keywords:** disease and pest resistance, rusts, pyramiding novel genes, marker-trait associations, candidate gene

## Abstract

Genetic resistance against biotic stress is a major goal in many wheat breeding programs. However, modern wheat cultivars have a limited genetic variation for disease and pest resistance and there is always a possibility of the evolution of new diseases and pests to overcome previously identified resistance genes. A total of 125 synthetic hexaploid wheats (SHWs; 2*n* = 6*x* = 42, AABBDD, *Triticum aestivum* L.) were characterized for resistance to fungal pathogens that cause wheat rusts (leaf; *Puccinia triticina*, stem; *P*. *graminis* f.sp. *tritici*, and stripe; *P*. *striiformis* f.sp. *tritici*) and crown rot (Fusarium spp.); cereal cyst nematode (*Heterodera* spp.); and Hessian fly (*Mayetiola destructor*). A wide range of genetic variation was observed among SHWs for multiple (two to five) biotic stresses and 17 SHWs that were resistant to more than two stresses. The genomic regions and potential candidate genes conferring resistance to these biotic stresses were identified from a genome-wide association study (GWAS). This GWAS study identified 124 significant marker-trait associations (MTAs) for multiple biotic stresses and 33 of these were found within genes. Furthermore, 16 of the 33 MTAs present within genes had annotations suggesting their potential role in disease resistance. These results will be valuable for pyramiding novel genes/genomic regions conferring resistance to multiple biotic stresses from SHWs into elite bread wheat cultivars and providing further insights on a wide range of stress resistance in wheat.

## 1. Introduction

Wheat (*Triticum aestivum* L.) is one of the most widely grown cereal crops with a production of more than 756 million tonnes in 2017–2018 [1] and it feeds more than one-third of the world’s population [2]. Global population is increasing at an alarming rate and is estimated to reach 9.8 billion by 2050 [3]. To meet global food demands, cereal production needs to be increased by 50% by 2030 [4]. However, biotic stresses caused by diseases and pests will impose significant constraints on agricultural production and productivity [5,6].

Wheat is grown in a wide range of environments and exposed to multiple diseases and pests throughout the growing season [7]. Biotic stresses observed in crops are divided into four groups: foliar and stem diseases, seed transmitted diseases, soilborne diseases, and pests. Foliar and stem diseases such as a leaf or brown rust (Lr; incited by *Puccinia triticina*), stem or black rust (Sr; incited by *P. graminis* f.sp. *tritici*), and stripe or yellow rust (Yr; incited by *P. striiformis* f.sp. *tritici*) are major threats to wheat production throughout the world [5,6]. Global wheat yield losses due to these rusts range from 20% to 100% [5,8,9,10] and were estimated at $5 billion per year since the 1990s [11]. Similarly, soilborne pathogens such as the cereal cyst nematode (CCNs; *Heterodera* spp.) cause significant cereal crop losses [12,13,14]. For instance, global crop losses due to nematodes have been estimated to be up to $80 billion [12]. Crown rot (Cr; caused by *Fusarium* spp.) is an important soilborne disease of wheat and severe Cr can cause significant yield losses in many wheat growing regions [15,16]. For example, the grain yield losses at different locations due to Cr have been reported to be up to 43% in Turkey [17], 46% in Iran [18], 61% in the United States [19], and 89% in Australia [20]. Hessian fly (HF; *Mayetiola destructor*), is one of the major destructive pests of wheat that causes grain yield loss of up to 80% [21] and may cause an average annual loss of 5% in the United States wheat production [22]. In Morocco, wheat grain yield loss due to Hessian fly was estimated to be up to 36% [23] and 42% [24]. The damage caused by diseases and pests can be reduced through crop rotation, cultural practices, and application of chemicals. Genetic resistance is the most economical, environment-friendly, and sustainable method of controlling crop losses. However, breeding for resistance to multiple diseases and pests requires identification of genetic sources of resistance and novel genes [7].

Synthetic hexaploid wheat (SHW; a cross between *T. turgidum* L. × *Aegilops tauschii* Coss.) is reported to have a novel source of genetic diversity for wheat genetic improvement especially for the D genome diversity [25,26] and is a potential source for resistance to abiotic [27] and biotic stresses [7,28,29]. For example, SHWs were found to have resistance to leaf rust [7,26,30], stem rust [7,31], stripe rust [7,31,32], Fusarium head blight (incited by *Fusarium graminearum*) [30], yellow spot (incited by *Pyrenophora tritici-repentis*) [7,31], Septoria glume blotch (incited by *Parastagonospora nodorum*) [7,31], Septoria leaf blotch (incited by *Zymoseptoria tritici*) [30,31], cereal cyst nematode [31], crown rot [7], root-lesion nematode (incited by *Pratylenchus thornei* and *P. neglectus*) [31], and Karnal bunt (incited by *Tilletia indica*) [32]. Additionally, SHWs have been reported to be resistant to multiple pests [7,28,30]. 

Currently, more than 110 leaf rust, 86 stem rust, and 83 stripe rust resistance genes have been reported in wheat or wild relatives, most conferring race-specific resistance [33]. Some of the important stem rust resistance genes are *Sr*2, *Sr*23, *Sr*24, *Sr*25, *Sr*31, *Sr*33, *Sr*35, *Sr*36, *Sr*38, *Sr*45, *Sr*50, *SrTmp*, and *Sr1RS*^Amigo^ [10]; the stripe rust resistance genes are *Yr2*, *Yr6*, *Yr7*, *Yr8*, *Yr9*, *Yr17,* and *Yr27;* and leaf rust resistance genes are *Lr9*, *Lr14a*, *Lr16*, *Lr17a*, *Lr21, Lr22, Lr24*, *Lr26,* *Lr32, Lr39*, and *Lr41* [8,10,34], where *Lr21, Lr22, Lr32, Lr39*, and *Lr41* were originated from *Ae. tauschii* [35]. To date, more than 35 Hessian fly resistance genes have been reported where *H*13, *H*22, *H*23, *H*24, *H*26, and *H*32 were originated from *Ae. tauschii* [33,36]. However, most of the resistance genes are short-lived due to the result of genetic recombination by sexual reproduction or somatic hybridization and mutation in the absence of alternative hosts [37], resulting in a modern variety with an inadequate level of insect-pest resistance. Therefore, exploiting genetic variation in SHWs for multiple biotic stresses and identifying genomic regions controlling their resistance is necessary for their utilization in an elite wheat breeding program. The objectives of this study were to (i) characterize unique sets of SHWs for resistance to CCN, Cr, HF, Lr, Sr, and Yr, (ii) identify lines resistant to multiple biotic stresses; (iii) identify genomic regions controlling their resistance using genome-wide association study (GWAS); and (iv) identify the potential genes underlying significant single nucleotide polymorphisms (SNPs). These data will provide useful information for pyramiding favorable alleles and multiple disease resistance genes from SHWs into elite bread wheat germplasm. 

## 2. Results and Discussion

### 2.1. Phenotypic Distribution of Disease and Pest

**Figure 1:**
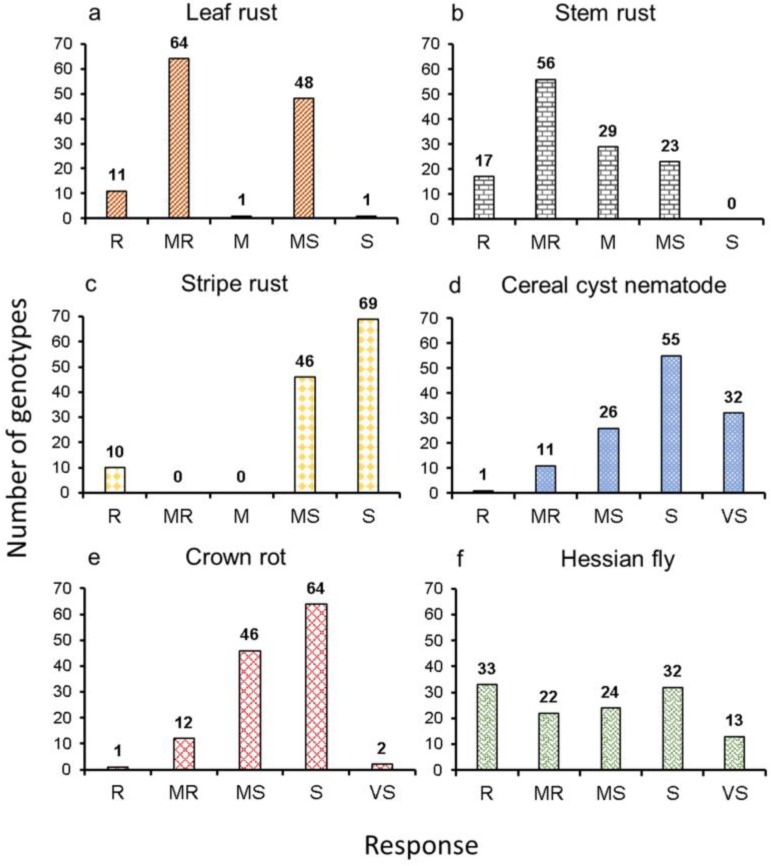
Frequency distribution of response to (**a**) leaf rust, (**b**) stem rust, and (**c**) stripe rust at the adult plant stage, (**d**) cereal cyst nematode, (**e**) crown rot, and (**f**) Hessian fly in 125 synthetic hexaploid wheat. R, Resistant; MR, Moderately resistant; M, Intermediate; MS, Moderately susceptible; S, Susceptible; and VS, Very susceptible.

A wide range of genetic variation for multiple biotic stresses was observed in 125 SHWs (Figure 1 and Supplementary Table S1). As expected, seedling and adult plant resistance screening for susceptible checks showed a moderately susceptible to very susceptible response and resistant checks showed a resistant to very resistant response, indicating that the screening methods were effective (Supplementary Table S1). More than 50% of the lines had a resistant (11) to moderately resistant (64) reaction to Lr at the adult plant stage (Figure 1a). For Lr seedling resistance screening, we identified 109 lines as immune (no symptoms) and 16 lines as very resistant. Of the 11 resistant lines at the adult plant stage, ten lines showed an immune response and one line showed a very resistant response at the seedling stage. These results indicated that 11 lines may possess major *Lr* resistance genes. 

Approximately 60% of the lines (73 lines) were resistant (17 lines) to moderately resistant (56 lines) to stem rust at the adult plant stage (Figure 1b). Seedling resistance screening against Sr identified 51, 52, 13, seven, and two lines as immune, very resistant, resistant, moderately resistant, and moderately susceptible, respectively against race TPMK (Supplementary Table S1). These results indicated that most of the lines may have *Sr* genes *Sr6*, *Sr9a*, *Sr9b*, or *Sr30* based on the TMPK race used. Of the 17 adult plant resistant lines, nine were immune, seven were resistant to very resistant, and one line was moderately resistant at the seedling stage, indicating that these lines may possess major *Sr* resistance genes. 

The adult plant stage Yr screening identified 10 resistant lines (Figure 1c). Similarly, seedling resistance screening identified one very resistant, 106 resistant, and 18 moderately resistant lines against the mixture of three *Pst* races (*Pstv*-14, *Pstv*-37, and *Pstv*-40) (Supplementary Table S1). This indicates that most of these synthetic genotypes may have *Yr* genes *Yr1*, *Yr5*, *Yr10*, *Yr15*, *Yr24*, *Yr17*, *Yr32*, *YrSP,* or *YrTye* or other unknown genes as they were resistant against three races (*Pstv*-14, *Pstv*-37, and *Pstv*-40). Of the 10 adult plant resistant lines, seven lines were resistant, and three lines were moderately resistant at the seedling stage, indicating that these lines may possess major *Yr* resistance genes.

We have also screened SHW lines for CCN, Cr, and HF resistance. This study identified one resistant and 11 moderately resistant lines for CCN (Figure 1d and Supplementary Table S1), one resistant and 12 moderately resistant lines for Cr (Figure 1e), and 33 resistant and 22 moderately resistant lines for HF (Figure 1f).

### 2.2. Genome-Wide Association Study

**Figure 2:**
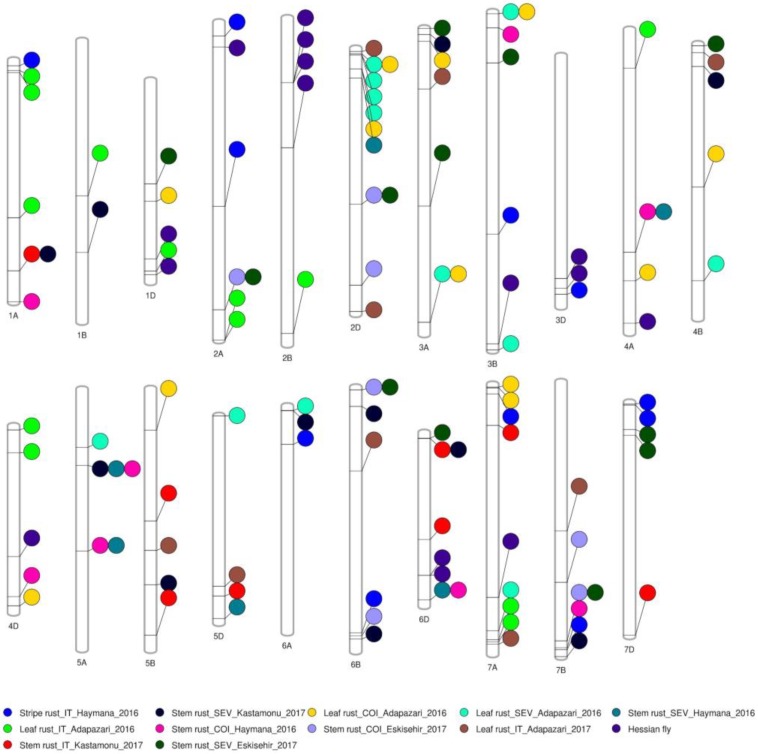
Significant marker-trait associations (*p* = 9.2 × 10^−5^ (−log_10_*p* = 4.04)) identified on each chromosome for leaf rust, stem rust, stripe rust, and Hessian fly from a genome-wide association study using 35,798 single nucleotide polymorphisms in 125 synthetic hexaploid wheats grown in 2016 and 2017 in Turkey (Adapazari, Eskisehir, Haymana, and Kastamonu) and in 2017 in Morocco (Settat; Hessian fly experiment). COI, Coefficient of infection; IT, Infection type; and SEV, Field disease severity.

A GWAS identified a total of 124 marker-trait associations (MTAs) for Hessian fly infestation (%), field-based infection type (IT) for Yr, and field disease severity (SEV), IT, and coefficient of infection (COI) for Lr and Sr (Figure 2 and Supplementary Table S2). The phenotypic variance explained (PVE) by these MTAs ranged from 0.3% to 27.4% (Supplementary Table S2). The MTAs identified in this study were year specific because of the significant influence of genotype × year interaction as can be seen by weak phenotypic correlation between years (*r* = 0.18; *p* = 0.06 to 0.42, *p* = 0.02). Also, we ran GWAS analysis for CCN and Cr, but we were unable to identify significant MTAs in our population; hence, the GWAS results for these traits will not be discussed further. Additionally, we did not run GWAS analysis on seedling rust resistance data because most of the genotypes were resistant and data were highly skewed (Supplementary Table S1).

At the adult plant stage for Lr, a total of 46 MTAs for SEV (12), IT (22), and COI (12) were identified on chromosomes 1A (3), 1B (1), 1D (2), 2A (2), 2B (1), 2D (8), 3A (4), 3B (3), 4A (2), 4B (3), 4D (3), 5A (1), 5B (2), 5D (2), 6A (1), 6B (1), 7A (6), and 7B (1) (Figure 2) and PVE by these MTAs ranged from 0.3% to 27.4% (Supplementary Table S2). To date, over 110 *Lr* resistance genes have been identified and they were distributed across all chromosomes in wheat except 5A and 6D [33]. Leaf rust resistance genes such as *Lr21, Lr22, Lr32, Lr39*, and *Lr41* were derived from *Ae. tauschii* [35]. The identification of 15 MTAs on the D genome clearly represent variation from the *Ae. tauschii* and show the potential of SHW for its utilization in a marker-assisted breeding program after being validated in an independent genetic background. This study identified three co-located MTAs (S2D_13778898, S3A_721353764, S3B_4553494) and a haplotype block (perfect linkage disequilibrium with *R*^2^ = 1) for Lr consisting of two SNPs (S2D_50105731 and S2D_50105753) of size 22 base pairs for COI and SEV on chromosomes 2D, 3A, and 3B (Supplementary Table S2). This was expected as these traits (SEV and COI) had a high correlation (ranging from 0.65 to 0.87, *p* < 0.001).

At the adult plant stage for Sr, a total of 52 MTAs for field SEV (29), IT (8), and COI (15) were identified on chromosomes 1A (3), 1B (1), 1D (1), 2A (2), 2D (4), 3A (3), 3B (2), 4A (2), 4B (2), 4D (1), 5A (5), 5B (3), 5D (2), 6A (1), 6B (5), 6D (6), 7A (1), 7B (5), and 7D (3) (Figure 2) with PVE ranging from 1.0 to 27.0% (Supplementary Table S3). To date, over 86 *Sr* genes have been identified, which were distributed across all chromosomes in wheat [33]. Several MTAs (17) identified on the D genome showed the potential of SHWs for improving Sr resistance in modern wheat cultivars. Additionally, this study identified seven co-located MTAs (S2D_381953220, S4A_545611013, S5A_208152827, S5A_440734987, S6B_7943502, S6D_456325441, and S7B_7067875454) for COI and SEV on chromosomes 2D, 4A, 5A, 6B, 6D, and 7B) and one co-located MTA (S6D_16745888) for IT and SEV on chromosome 6D. Identification of co-located MTAs was supported by the high correlation among these traits (*r* > 0.64, *p* < 0.001).

Similarly, at the adult plant stage for Yr, a total of 11 MTAs for IT were identified on chromosomes 1A (1), 2A (2), 3B (1), 3D (1), 6A (1), 6B (1), 7A (1), 7B (1), and 7D (2) (Figure 2) with PVE ranging from 0.4% to 15.5% (Supplementary Table S3). To date, over 83 *Yr* resistance genes have been reported, which were distributed across all chromosomes in wheat [33]. Most of the genes are race-specific and are often short-lived due to the frequent change in the pathogen population [32]. The MTAs identified in the current study showed the quantitative nature of the resistance to Yr in SHWs. Previous studies have identified MTAs for Yr on chromosomes 2A, 3B, 6A, and 7B in an association mapping panel of 181 SHWs [32]. The MTAs identified on the D genome (chromosomes 3D and 7D) showed the potential of SHWs for improving Yr resistance in modern wheat cultivars.

For HF, 15 MTAs were identified on chromosomes 1D (2), 2A (1), 2B (4), 3B (1), 3D (2), 4A (1), 4D (1), 6D (2), and 7A (1) (Figure 2) and PVE by these MTAs ranged from 0.4% to 15.5% (Supplementary Table S3). A previous study comprised of 134 wheat genotypes (landrace, SHW, and elite germplasm) had identified seven MTAs responsible for HF resistance on chromosomes 1B, 2D, 3A, 3D, 5D, and 7D [36]. The 13 MTAs identified for HF resistance in this study have not been previously reported and they are potentially novel MTAs responsible for HF resistance.

### 2.3. Genes Underlying Marker-Trait Associations and Their Functional Annotations

The functional annotation of genes underlying MTAs was available through IWGSC RefSeq v1.0 annotations. A total of 33 of 124 MTAs were found within genes and of these, 16 were present in seven genes whose functional annotations suggested their involvement in disease resistance in wheat based on previous literature (Table 1 and Supplementary Table S3). The identification of underlying genes with annotations related to the trait provides further reliability for the MTAs identified. For instance, four genes (TraesCS4D01G096900: Lr, TraesCS7A01G517300: Lr, TraesCS4B01G007000: Sr, and TraesCS3A01G495400: Lr) were annotated as the leucine-rich repeat protein family, which was previously reported to have a potential role in Yr [38,39] and Sr resistance [40]. Similarly, five genes (TraesCS2A01G076800: Yr, TraesCS7B01G473300: Yr, TraesCS1A01G325300: Sr, TraesCS6B01G394600: Yr, and TraesCS5B01G199700: Sr) were annotated as the F-box family protein, which were reported to have potential role in Lr [41], Sr [42], and Yr [43] resistance in wheat. These two classes of genes are interesting genes for future study in wheat as they were also reported to be involved in abiotic stress tolerance such as drought [27,44,45]. However, further validation studies are needed for the MTAs identified and underlying gene functions prior to their utilization in a marker-assisted selection program. Additional examples where the underlying genes had annotations matching the trait of interest are provided in Table 1.

### 2.4. Multiple Stress Resistant Lines

**Figure 3:**
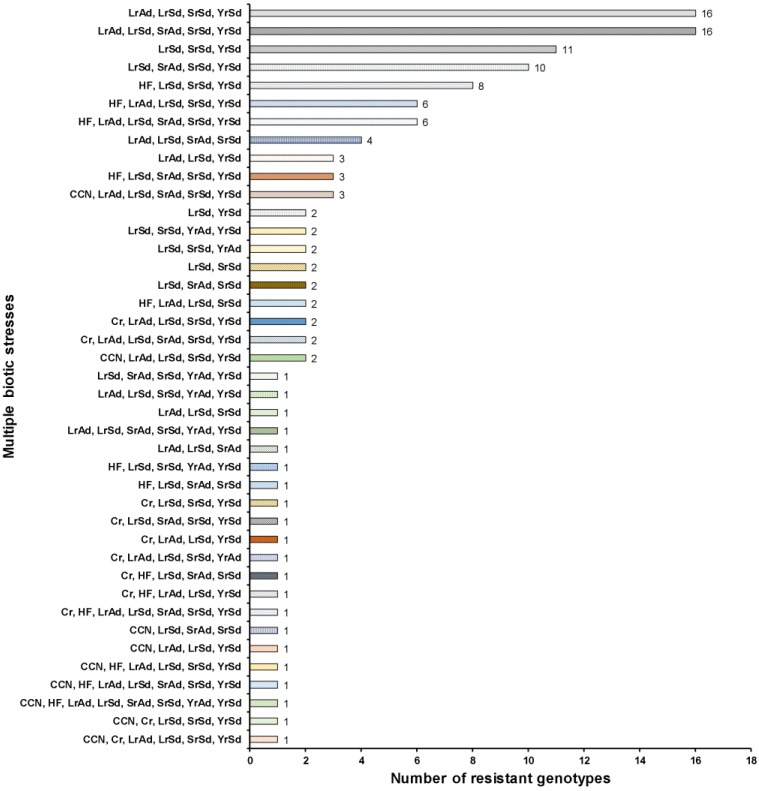
Number of synthetic hexaploid wheat lines showing resistance to multiple biotic stresses. CCN, cereal cyst nematode; Cr, crown rot; HF, Hessian fly; Lr, leaf rust; Sr, stem rust; Yr, stripe rust; Sd, seedling stage; and Ad, adult stage.

There were six biotic stresses (three rusts assessed at both seedling and adult plant stages, CCN, Cr, and HF) assessed to identify multiple stress resistant lines. All the SHW lines showed resistance to multiple biotic stresses ranging from two to five biotic stresses (Figure 3 and Supplementary Table S1). Specifically, 14, 67, 38, and 6 lines showed resistance against two, three, four, and five biotic stresses, respectively (Figure 3). From this study, we can recommend 17 lines (15.3% of the total resistant lines) that were resistant to more than two stresses and had a large number of favorable alleles (alleles that increased its resistance against multiple stresses based on significant markers identified in GWAS) conferring resistance (Supplementary Table S4). These lines can be used as parents in breeding programs with the goal of reducing disease and pest damage. The results indicated the usefulness of these lines for pyramiding multiple genes for durable resistance to multiple diseases and pests.

## 3. Materials and Methods

### 3.1. Genetic Resources

A total of 125 synthetic hexaploid wheat (110 winter synthetics and 15 spring synthetics) lines derived from crosses between seven durum (*T. turgidum*) parents and 25 different *Ae. tauschii* accessions were used in this study (Supplementary Table S1). These lines were distributed by the International Winter Wheat Improvement Program (http://www.iwwip.org) and details were provided previously [25,27,50].

### 3.2. Evaluation of Disease and Insect Resistance

#### 3.2.1. Seedling Resistance Screening for Leaf, Stem, and Stripe Rusts

Seedlings of 125 SHWs were evaluated in the greenhouse for Lr disease reaction using mixed spores collected from Nebraska wheat fields, against Sr race “TPMK” (Isolate 74MN1409, avirulence/virulence formula *Sr6*, *Sr9a*, *Sr9b*, *Sr24*, *Sr30*, *Sr31*, *Sr38*, *Sr1A.1R./Sr5*, *Sr7b*, *Sr8a*, *Sr9a, Sr9d, Sr9e*, *Sr10, Sr11*, *Sr17, Sr21, Sr36, SrMcN*, *SrTmp*) [51], and against Yr using a mixture of three races (*Pstv*-14: avirulence/virulence formula on *Yr* genes *Yr5*, *Yr10*, *Yr15*, *Yr24*, *Yr32*, *YrSP/Yr1*, *Yr6*, *Yr7*, *Yr8 Yr 9*, *Yr17*, *Yr27*, *Yr43*, *Yr44*, *YrTr1*, *YrExp2*, *YrTye*; *Pstv*-37: avirulence/virulence formula on *Yr* genes *Yr1*, *Yr5*, *Yr10*, *Yr15*, *Yr24*, *Yr32*, *YrSP*, *YrTye/Yr6*, *Yr7*, *Yr8 Yr 9*, *Yr17*, *Yr27*, *Yr43*, *Yr44*, *YrTr1*, *YrExp2*; and *Pstv*-40: avirulence/virulence formula on *Yr* genes *Yr1*, *Yr5*, *Yr15*, *Yr17*, *YrSP*, *YrTye/Yr6*, *Yr7*, *Yr8*, *Yr9*, *Yr10*, *Yr24*, *Yr27*, *Yr32*, *Yr43*, *Yr44*, *YrTr1*, *YrExp2*). The experimental design used in these studies was a randomized complete block design with six replications. Seedling inoculation procedures were performed using previously described protocols for Lr (http://plantsciences.montana.edu/facultyorstaff/faculty/huang/Rust%20Inoculation%20Protocols.pdf), Yr (https://s3.wp.wsu.edu/uploads/sites/2151/2015/12/Greenhouse-Protocols-for-Testing-and-Handling-Stripe-Rust.pdf), and Sr [51]. At 14–18 days after inoculation, leaf and stem rust infection types were scored based on a scale of 0–4 [52,53]. The infection type score of 0 was considered as immune (I) or as very resistant, 1 as resistant (R), 2 as moderately resistant (MR), 3 as moderately susceptible (MS), and 4 as susceptible (S). Stripe rust infection types were scored based on a scale of 0 (very resistant) to 9 (very susceptible) as described previously [54]. For Sr resistance screening, the cultivar “Goodstreak” was used as a resistant check whereas “Cheyenne” and “McNair 701” were used as susceptible checks. For Lr resistance screening, the cultivar “Karahan” was used as a resistant check whereas “Goodstreak” and “McNair 701” were used as susceptible checks. For Yr resistance screening, the cultivar ‘Summit 515’ was used as a resistant check whereas “Morocco” was used a susceptible check.

#### 3.2.2. Adult Plant Resistance Screening for Leaf, Stem, and Stripe Rusts

For the adult plant rust screening, all the genotypes were tested under field conditions for Lr, Sr, and Yr resistance in an augmented design with replicated checks (“Gerek” and “Karahan”) in two rows (distance between rows: 22.5 cm) of a meter-long plot. Leaf rust was tested under natural conditions in Adapazarı, Turkey in the 2016 and 2017 growing seasons. Stem rust was tested against artificial inoculation of race “TTTTF” (avirulent on: *Sr24* and *Sr31*; virulent on: *Sr5*, *Sr6*, *Sr7b*, *Sr8a*, *Sr9a*, *Sr9b*, *Sr9d*, *Sr9e*, *Sr9g*, *Sr10*, *Sr11*, *Sr17*, *Sr21*, *Sr30*, *Sr36*, *Sr38*, and *SrMcN*) at Haymana in Turkey in 2016 under natural conditions using natural races in Kastamonu in Turkey in 2016 and 2017, Eskisehir in Turkey, and at Njoro, Kenya in 2017. Similarly, Yr was tested against the artificial inoculation of “Warrior race” (avirulent on: *Yr8* and *Yr27*; virulent on: *Yr1*, *Yr2*, *Yr3*, *Yr4*, *Yr6*, *Yr7*, *Yr9*, *Yr17*, *Yr25*, *Yr32*, *YrSp*, *YrAvs*, and *YrAmb*) under field conditions at Haymana research station in Turkey in 2016, whereas in 2017, the genotypes were tested under natural conditions using natural races of Yr at Kenya Agricultural and Livestock Research Organization (KALRO) in Njoro, Kenya. Yellow rust disease severity on the flag leaf was recorded three times during the growing season and the final score was taken as a representative score for the analysis. “Gerek” was a susceptible check and “Karahan” was a resistant check for the Lr, Sr, and Yr (Warrior race) used in this study. Rust disease intensities at the adult plant stage were recorded using a modified cobb scale [55] based upon severity (percentage of the plant infected) and response (a type of disease reaction). Field disease severity (SEV) was recorded as a percentage using the following scores: Trace, 5, 10, 20, 40, 60, and 100% infection, whereas a disease response or an infection type (IT) was recorded by dividing the infection type into six groups: 0, resistant (R), moderately resistant (MR), moderately resistant to susceptible (M), moderately susceptible (MS), and susceptible (S) [55]. An average coefficient of infection (COI) was calculated as the multiplication of severity and assigned a constant value based on IT. Constant values were 0.2, 0.4, 0.6, 0.8, and 1.0 for field responses of R, MR, MR–MS (M), MS, and S genotypes, respectively.

#### 3.2.3. Cereal Cyst Nematode Resistance Screening

The cereal cyst nematode resistance screening was carried out at the Soil Borne Diseases Program at International Maize and Wheat Improvement Center (CIMMYT)-Turkey facilities located at the Transitional Zone Agriculture Research Institute (TZARI) Eskisehir, Turkey. The screening was performed in 2016 and 2017 in a randomized complete block design with three replications in the growth chamber as per [14]. Briefly, the population of *H. filipjevi* was collected from a field in Yozgat, Turkey and freshly hatched second stage juveniles (J2s) were used as inoculum in the screening tests. Three replicates per genotype were inoculated after emergence with 400 freshly hatched J2 by making three holes around the stem base under controlled conditions. Nine weeks after inoculation, cysts from both root and soil extractions were counted under a stereomicroscope. The mean number of cysts was recorded and classed into five groups: Resistant (R), equal or fewer cysts than in a known resistant check; moderately resistant (MR), slightly more cysts than in a resistant check; moderately susceptible (MS), slightly more cysts than in an MR check whereas less than in a susceptible (S) check; S, cyst number similar to S check; and highly susceptible (VS), more cysts than in the S check [14]. Wheat cultivars “Katea”, “Sonmez”, and “Yelken” were used as resistant checks whereas “Bezostaja” and “Kutluk” were used as susceptible checks for CCN.

#### 3.2.4. Crown Rot Resistance Screening

Crown rot resistance screening was performed in a randomized complete block design with three replicates under greenhouse conditions at the Transitional Zone Agricultural Research Institute, Eskisehir, Turkey in 2016 and 2017. The crown rot resistance screening protocol used in this study was provided in detail in Erginbas-Orakci et al. [56]. In brief, the pathogenic *F. culmorum* Isolate 18 and Isolate 41 were isolated from a mature wheat crowns near Usak city in the Aegean region and Konya, Central Anatolia Plateau of Turkey, respectively. Three replicates of each genotype were grown in a plastic tube (3 cm diameter × 12.5 cm in length) in the greenhouse for about two months with a day/night photoperiod of 16/8 h at a temperature of 25/15 °C and a relative humidity of 60/80%. Around 0.5 g fungus colonized wheat bran was added to the tubes at the time of seed sowing. Around six weeks after inoculation (Zadoks growth stage 14), plants were scored using a 1–5 scale: 1, Resistant; 2, Moderately Resistant; 3, Moderately Susceptible; 4, Susceptible; and 5, Very Susceptible [56]. Wheat cultivars “Altay”,”Carisma”, “Katea”, “Mace”, “Spitfire”, “Suntop”, “Wallup”, “249”, were used as R–MR checks; “Scout”, “Sonmez”, and “Sunco” were used as MR–MS checks; “Bezostaya”,”Ega wylie”, “Emu Rock”, and “Janz” were used as MS checks; whereas “Dumlupinar”, “Kiziltan”, “Kutluk”, “Puseas”, “Seri”, and “Suzen97” were used as susceptible checks for Cr.

#### 3.2.5. Hessian Fly Resistance Screening

The Hessian fly resistance screening using a population from Morocco was performed in 2017 in the greenhouse (20 °C and 70% relative humidity) at the International Center for Agricultural Research the Dry Areas (ICARDA) in Settat, Morocco [28]. A genotype that was resistant to this population may have Hessian fly resistance genes *H5*, *H11*, *H13*, *H14*, *H15*, *H21*, *H22*, *H23*, *H25*, or *H26* [28]. The detailed protocol for Hessian fly resistance screening used in this study was provided previously [57]. Briefly, 10 plants per genotype were grown in a greenhouse flat (54 × 36 × 8 cm). Then the seedlings at the one leaf stage were placed under a cheesecloth tent along with infested plants containing mature Hessian fly pupae from which adult Hessian flies emerged and infected the seedling plants. Plant reactions to larval feeding were determined after 20 days. Susceptible genotypes were stunted, dark green, and contained live larvae whereas resistant genotypes were not stunted, light green, and contained dead or live larvae. Percentage of resistant plants was calculated as the number of resistant plants divided by a total number of plants evaluated. The percentage score was divided into five groups: 0%, very susceptible (VS); 10–40%, susceptible; 50–70%, moderately susceptible; 80–90% moderately resistant (MR); and 100% as resistant (R) genotype [57]. Wheat cultivar ‘Nesma’ was used as a susceptible check [57] and resistant plants were checked (under a microscope; 40x) for the presence of dead larvae to confirm the antibiosis effect [36].

### 3.3. Genotyping and SNP Discovery

The details on genotyping and SNP discovery were described previously [25,27]. Briefly, the genotyping was performed using genotyping-by-sequencing (GBS) approach and SNP discovery was made using TASSEL v. 5.2.40 GBS v2 Pipeline [58] with physical alignment to the Chinese Spring genome sequence, RefSeq v1.0 [59]. The SNPs with minor allele frequency less than 5% and missing information greater than 20% were excluded [50]. All genotypes in this study met the filtering criteria of missing sites less than 20% and were retained in this study [25,27].

### 3.4. Population Structure and Genome-Wide Association Study

The population structure analysis of 125 SHWs used in this study was performed using a Bayesian clustering algorithm in STRUCURE v.2.3.4 [60] and principal component analysis in TASSEL [61], and has been described previously [29,50]. A GWAS was conducted using 35,798 GBS-derived SNPs and best linear unbiased estimates (BLUEs) of HF infestation score, CCN score, Cr score, and field-based rust SEV, IT, and COI for each location and year. BLUEs were estimated using PROC MIXED in SAS 9.4 (SAS Institute Inc., Cary, NC) by assuming genotype as a fixed effect and replication/block as a random effect. A multi-locus mixed linear model using FarmCPU (Fixed and random model Circulating Probability Unification) algorithm with the coefficient of ancestries of the first three population structure subgroups as covariates and FarmCPU calculated kinship [62] as a random effect were implemented in the Memory-efficient, Visualization-enhanced, and Parallel-accelerated Tool (MVP R software package—https://github.com/XiaoleiLiuBio/MVP) for GWAS. The marker-trait associations (MTAs) were selected with a genome-wide significance threshold level of *p* = 9.18 × 10^−5^ (−log_10_*p* = 4.04) considering the deviation of the observed test statistics value from the test statistics values in the quantile–quantile plots [27]. Haplotype block analysis was performed using default parameters settings of Haploview software (https://www.broadinstitute.org/haploview/haploview). The gene underlying MTAs and their annotation was retrieved from the IWGSC RefSeq v1.0 annotation. The potential candidate genes associated with the trait of interest were further examined using published literature.

## 4. Conclusions

The present study identified SHWs resistant to diseases and pests that can be used for breeding for resistance to multiple biotic stresses. Additionally, this study identified several lines having resistance to multiple rusts (Lr, Sr, and Yr) at both the seedling and adult plant stage. Resistance genes from these lines can be transferred to bread wheat cultivars using back-crossing.

From GWAS, this study identified 124 genomic regions associated with various diseases that can be used in a marker-assisted breeding program upon validation in an independent population. Furthermore, several genes in those significant genomic regions had gene annotations suggesting their involvement in disease resistance, which provided confidence on the reliability of the identified genomic regions. Furthermore, this study identified several MTAs (42) on the D genome which demonstrates the potential of SHWs to expand the genetic resources of the D genome and for their utilization in wheat breeding programs. Finally, this study also provided information towards further understanding of resistance to multiple biotic stresses in wheat.

## Figures and Tables

**Table 1:** List of potential candidate genes found in regions identified by marker-trait associations for disease resistance, favorable allele (bold), and SNP effects in 125 SHWs.

Trait ^a^	SNP_ID ^b^	PVE ^c^	Allele ^d^	Effect	−Log_10_(*p*)	Gene-ID	Potential Candidate Gene	Trait ^e^	References ^f^
Yr	S3B_544810753	6.4	**G**/A	−0.09	8.21	TraesCS3B01G338200	Calcium-binding protein	Yr	[46]
Lr	S4D_73425349	18.5	**C**/T	0.27	10.14	TraesCS4D01G096900	Leucine-rich repeat transmembrane neuronal protein 3	Yr, Sr	[38,39,40]
Sr	S7B_706787545	15.1	**A**/G	15.54	7.95	TraesCS7B01G441400	ARM repeat superfamily protein	Tanspot	[47]
Yr	S2A_34483654	12.4	**T**/G	−0.13	9.63	TraesCS2A01G076700	Elongation factor 1 alpha a	Yr	[43]
Yr	S2A_34483654	12.4	**T**/G	−0.13	9.63	TraesCS2A01G076800	F-box protein	Lr, Sr, Yr	[41,42,43]
Yr	S7B_730117633	3.8	**C**/A	−0.09	7.54	TraesCS7B01G473300	F-box protein	Lr, Sr, Yr	[41,42,43]
Sr	S1A_516079891	9.9	**G**/C	−0.09	6.29	TraesCS1A01G325300	F-box protein	Lr, Sr, Yr	[41,42,43]
Yr	S6B_669910684	0.9	**C**/G	0.15	8.03	TraesCS6B01G394600	F-box protein	Lr, Sr, Yr	[41,42,43]
Sr	S5B_360428982	10.8	**T**/C	−0.07	6.95	TraesCS5B01G199700	F-box protein	Lr, Sr, Yr	[41,42,43]
Lr	S5A_158912458	8.7	**G**/A	−5.51	4.96	TraesCS5A01G103000	F-box protein	Lr, Sr, Yr	[41,42,43]
Lr	S7A_701569874	8.5	**A**/G	0.15	8.60	TraesCS7A01G517300	Leucine-rich repeat protein kinase family protein	Lr, Sr, Yr	[41,42,43]
Sr	S4B_4791631	3.0	**G**/C	−6.10	7.43	TraesCS4B01G007000	Leucine-rich repeat receptor-like protein kinase family protein	Sr, Yr	[38,39,40]
Sr	S2D_381953220	8.4	**G**/T	8.33	7.48	TraesCS2D01G299700	Peroxidase	Sr, Tanspot	[47,48]
Lr	S3A_721353764	14.6	**G**/T	3.51	7.78	TraesCS3A01G495400	Leucine rich repeat N-terminal domain	Sr, Yr	[38,39,40]
Sr	S1A_592532938	6.4	**G**/A	−7.50	5.54	TraesCS1A01G444500	Leucine rich repeat N-terminal domain	Sr, Yr	[38,39,40]
Lr	S2D_644835165	1.4	**G**/T	0.07	5.89	TraesCS2D01G586500	WAT1-related protein/EamA-like transporter family	Fungal pathogen	[49]
Lr	S7A_708435571	19.4	**T**/C	−0.18	8.20	TraesCS7A01G526900	WAT1-related protein/EamA-like transporter family	Fungal pathogen	[49]

^a^ Lr, leaf rust; Sr, stem rust; Yr, stripe rust. ^b^ S+chromosome_position in bp, ^c^ PVE, phenotypic variance explained (%), ^d^ Allele that is in bold text is the favorable allele that increases the disease resistance, ^e^ Trait identified in the past^, f^ References for the association of annotation with traits.

## Supplementary Materials

Supplementary Materials can be found at https://www.mdpi.com/1422-0067/20/15/3667/s1.

## Abbreviations

SHW	Synthetic hexaploid wheat
CCN	Cereal cyst nematode
Cr	Crown rot
HF	Hessian fly
Lr	Leaf rust
Sr	Stem rust
Yr	Stripe rust
GBS	Genotyping-by-sequencing
SNP	Single nucleotide polymorphism
GWAS	Genome-wide association study
MTA	Marker-trait association
BLUE	Best linear unbiased estimate
PVE	Phenotypic variance explained
SEV	Field disease severity
COI	Coefficient of infection
IT	Infection type
